# Case series of progressive familial intrahepatic cholestasis type 3: Characterization of variants in *ABCB4* in China

**DOI:** 10.3389/fmed.2022.962408

**Published:** 2022-12-08

**Authors:** Jinlin Cheng, Ling Gong, Xiaoxiao Mi, Xiangyan Wu, Jun Zheng, Wenjun Yang

**Affiliations:** ^1^State Key Laboratory for the Diagnosis and Treatment of Infectious Diseases, National Clinical Research Center for Infectious Diseases, Collaborative Innovation Center for Diagnosis and Treatment of Infectious Diseases, The First Affiliated Hospital, College of Medicine, Zhejiang University, Hangzhou, China; ^2^Department of Infectious Diseases, The Affiliated Hospital of Hangzhou Normal University, Hangzhou, China; ^3^Department of Translational Medicine Platform, The Affiliated Hospital of Hangzhou Normal University, Hangzhou, China; ^4^Department of Pathology, The Affiliated Hospital of Hangzhou Normal University, Hangzhou, China

**Keywords:** PFIC, PFIC3, *ABCB4*, cholestasis, MDR3

## Abstract

**Objective:**

To improve the accuracy of the diagnosis of familial progressive intrahepatic cholestasis type 3 (PFIC3, https://www.omim.org/entry/602347).

**Materials and methods:**

Between September 2019 and March 2021, we recruited four patients with PFIC3 from two liver centers in East China. Molecular genetic findings of ATP-binding cassette subfamily B member 4 [ATP binding cassette transporter A4 (*ABCB4*), https://www.omim.org/entry/171060] were prospectively examined, and clinical records, laboratory readouts, and macroscopic and microscopic appearances of the liver were analyzed.

**Results:**

Four patients experienced cholestasis, mild jaundice, and elevated levels of serum direct bilirubin, γ-glutamyltransferase, or total bile acids. All patients had moderate-to-severe liver fibrosis or biliary cirrhosis, and their liver biopsy specimens stained positive with rhodamine. Molecular immunohistochemistry revealed reduced or absent MDR3 expression in all liver specimens. A novel mutation of *ABCB4* (c.1560 + 2T > A) was identified in patients with PFIC3, which is of high clinical significance and may help understand mutant *ABCB4* pathogenesis.

**Conclusion:**

MDR3 immunohistochemistry and molecular genetic analyses of *ABCB4* are essential for the accurate diagnosis of PFIC3.

## Introduction

Progressive familial intrahepatic cholestasis (PFIC) is a genetic disorder with autosomal recessive inheritance. It primarily manifests as jaundice and itchy skin and then progresses from persistent cholestasis to cirrhosis and liver failure before late childhood (1). Thus far, PFIC has been reported in Caucasian, Asian, and African patients. The estimated incidence of PFIC ranges from 1 per 50,000 to 1 per 100,000 worldwide (2). Based on the gene carrying the mutation, PFIC is currently identified to result from mutations in six genes, namely *ATP8B1*, ATP-binding cassette subfamily B member 11 (*ABCB11*), ATP binding cassette transporter A4 (*ABCB4*), *TJP2*, *NR1H4*, and *MYO5B*. There are three classic types of PFIC, referred to as PFIC1, PFIC2, and PFIC3, which result from a defect in a biliary protein involved in bile formation and circulation in the liver (3). PFIC1 and PFIC2 represent two-thirds of PFIC cases, whereas PFIC3 represents one-third of PFIC cases (4–8).

PFIC1 and PFIC2 show clinical symptoms in the neonatal period and rapidly progress to liver failure but with normal or reduced GGT levels. Conversely, PFIC3 is clinically present in adolescence or adulthood, progresses slowly, and GGT levels are elevated. PFIC3 is caused by mutations in the *ABCB4* gene (The ATP binding cassette subfamily B member 4), which result in multidrug resistance-associated protein 3 (MDR3) to be truncated, unstable, misfolded, or impaired during its transport (9, 10). *ABCB4* gene is located on human chromosome 7q21, with a total of 75504 bases and 28 exons, of which 27 exons have coding sequences and the first exon has a 26-bp non-translated region (11).

MDR3 protein is a protein encoded by the *ABCB4* gene. It plays an important role in normal bile formation and is distributed in the surface of cholangiole of hepatocytes. It has 12 transmembrane regions; the N-terminus and C-terminus are on the inner side of the hepatocyte membrane, which is actually phosphatidyl transferase enzyme, and mediates the transport of phosphatidylcholine (PC) to bile from the inside of the hepatocyte membrane to the outside. The phospholipids in normal bile can form stable mixed particles (mixed micelles) in appropriate proportions with bile salts. They emulsify bile salts and avoid them from precipitating to form crystals, thus protecting bile duct epithelial cells and promoting cholesterol secretion (12). Disease-specific *ABCB4* gene polymorphisms have been described in some intrahepatic cholestasis diseases, suggesting decreased MDR3 expression and function (12).

Recent studies have shown that the *ABCB4* gene mutation types include truncated mutations, missense mutations, frameshift mutations, nonsense mutations, and splicing site mutations. Some of these may clinically manifest as various chronic progressive lesions (13). Some mutant heterozygotes usually have normal clinical manifestations; however, they can still exhibit various symptoms caused by low MDR3 function when induced by certain factors, such as drugs.

Variations introducing premature stop codons cause a defect in protein expression; however, more than 70% of disease-causing *ABCB4* variations are missense variations, and the ways in which such variations affect *ABCB4* are generally unknown (14). Theoretically, missense mutations could affect the protein in several ways, either by modulating its expression at the canalicular membrane or by impairing its activity.

At present, clinical examination can identify *ABCB4* gene deficiency-related diseases based on the patient’s medical history, blood biochemical examination, liver tissue biopsy, liver imaging, and other auxiliary tests; however, the method commonly used to confirm *ABCB4* deficiency is genetic sequence detection and MDR3 immunohistochemical staining of liver tissue slices. Notably, gene polymorphisms of *ABCB4* may affect the function of MDR3 proteins. MDR3 immunohistochemical staining methods are simple, but whether the staining results are consistent with genetic mutations requires further exploration.

This study was aimed at improving the diagnosis of PFIC3 by observing whether the expression of MDR3 staining in liver puncture tissue sections was consistent with the gene mutation and analyzing clinical, pathologic, genetic, and clinical features of four cases of Chinese patients.

## Patients and methods

### Patients

Between September 2019 and March 2021, four patients with PFIC3 were recruited from two liver centers in East China, including three patients from the Affiliated Hospital of Hangzhou Normal University and one patient from the First Affiliated Hospital of Zhejiang University. Cholestasis was defined as follows: (1) γ-glutamyl transpeptidase (GGT) levels higher than three times the upper limit of normal (ULN) and serum alkaline phosphatase levels higher than 1.5 times the upper limit of normal (ULN); (2) direct bilirubin (DBil) levels higher than 17. 1 μmol/L and serum total bilirubin (TBil) levels lower than 85.0 mol/L; or (3) DBil accounting for > 20% of TBil when TBil is ≥ 85.0 mol/L (15).

In this study, PFIC3 presented as (1) the appearance of clinical symptoms of cholestasis, including mild yellow dye in the skin and sclera, (2) elevated serum DBil, GGT, or total bile acid (TBA) levels, (3) the presence of the *ABCB4* gene and its mutants, and (4) cholestasis identified as not having any cause besides the aforementioned ones through ultrasound B, isotope liver scan, and genetic metabolic disease screening. Clinical courses and molecular genetic results for *ABCB4* were examined retrospectively and prospectively for patients with PFIC3 for laboratory values, macroscopic and microscopic liver characteristics, and molecular genetic characteristics of the liver. Our study protocol was compliant with ethical guidelines of the Declaration of Helsinki of 1975 (2004 revision), the Ethical Committee of the Affiliated Hospital of Hangzhou Normal University (approval number: 20211223090600282150), and the First Affiliated Hospital of Zhejiang University (approval number: 2021IIT0286). Considering the retrospective design of the study and anonymity of the patients, the need for obtaining written informed consent from the patients was waived by the ethics committees.

### Histopathology of the liver

Patients with PFIC3 had liver biopsy specimens collected during causal evaluations of their cholestasis. To determine basic histologic characteristics and check for collagen and copper granule deposits in hepatocytes, 4-μm sections of formalin-fixed, paraffin-embedded blocks were cut and stained with hematoxylin and eosin, Masson’s trichrome, and rhodamine. Epithelial cells of the bile duct were immunohistochemically stained for CK7. Immunohistochemical staining was also performed for MDR3 (clone EPR23697-35, dilution 1:3000; Abcam, London, United Kingdom). The immunohistochemistry of MDR3 was considered positive only if it stained in a canalicular pattern. Two pathologists evaluated all biopsy specimens.

### Molecular genetic analysis of *ABCB4*

Whole-exome sequencing (WES) and bioinformatics were used to detect and analyze genetic variations in *ABCB4* using the Illumina NovaSeq 6000 sequencing system. Although exons account for only 1–2% of the human genome, they contain 85% of the pathogenic variants. Our sequencing was based on a standardized target area capture sequence platform. The sequencing quality control indicators are the average coverage rate of 99.11% and an average sequencing depth of 158.81 ×.

To describe the nucleotide substitutions, we used the NCBI transcript sequence of *ABCB4*: NM_000443.3, which contains all candidates for single nucleotide variants and small indels. We used a public database (gnomAD database) to estimate the prevalence of mutations in the *ABCB4* gene in the East Asian populations and used the ClinVar database and Human Gene Mutation Database of 2021 to predict the correlation between *ABCB4* gene mutation and disease occurrence. Then, we predicted the function of proteins (harmful or harmless) coded by genes with missense mutation of the novel variants using the Sorting Intolerant From Tolerant (SIFT) program and Polymorphism Phenotyping v2 (PolyPhen-2). The pathogenicity of the detected mutations was evaluated principally on the basis of the relevant guidelines of the American College of Medical Genetics and Genomics (16).

## Results

The study included four patients (one man and three women) with a median age at referral of 26 years (range, 17–45 years; Table 1). Cholestasis presented with jaundice as the first clinical sign in all patients, except for patient 2, who presented with thrombocytopenia as the first clinical sign, which was identified in routine medical examination findings. By appropriately investigating these causes, other common causes of cholestasis, including biliary atresia, congenital biliary dilation, viral hepatitis, and other metabolic disorders, such as citrin deficiency, benign recurrent intrahepatic cholestasis, and bile acid synthesis defects, were excluded. Currently, no commercial tests are available in China for testing urinary coproporphyrin isomers. In all patients who had fasted for 3 h, abdominal ultrasound revealed liver cirrhosis and different manifestations of portal hypertension, including splenomegaly, esophageal varicose veins, thrombocytopenia, and ascites (Table 1).

**TABLE 1:** Clinical characteristics and laboratory findings.

Clinical information	Progressive familial intrahepatic cholestasis type 3 (PFIC3)			
	
	Case 1	Case 2	Case 3	Case 4
Gender	Female	Female	Male	Female
Age (years)*	24	32	33	45
Cholelithiasis	No	Gallbladder and common	Common bile	Common bile
		bile duct calculi	duct calculi	duct calculi
Jaundice	Yellow staining of the sclera		Yellow staining of the sclera, yellow urine	Yellow urine
TBil (μmol/L)	62.9	34.4	219.3	22.4
DBil (μmol/L)	22.1	7.6	108.7	20.2
TBA (μmol/L)	63.2	84.7	/	4.4
ALT(U/L)	45	64	74.7	12
AST(U/L)	60	51	108.8	19
ALP(U/L)	159	174	160	234
GGT(U/L)	104	211	196	126
Total Cholesterol	2.01	5.21	4.27	5.21
Triglycerides	0.52	0.75	2.37	2.12
HDL-C	0.74	1.86	0.7	0.61
Portal hypertension	Splenomegaly, varicose veins of the esophagus	Splenomegaly, thrombocytopenia		Splenomegaly, varicose veins of the esophagus, ascites

TBil, total bilirubin; DBil, direct bilirubin; TBA, total bile acid; ALT, alanine aminotransferase; AST, aspartate aminotransferase; ALP, alkaline phosphatase; GGT, γ-glutamyl transpeptidase; HDL-C, high-density lipoprotein-cholesterol.

*Age is the actual age at referral.

All patients had abnormal liver functions suggestive of cholestasis (Table 1).

### Histopathological findings

**FIGURE 1:**
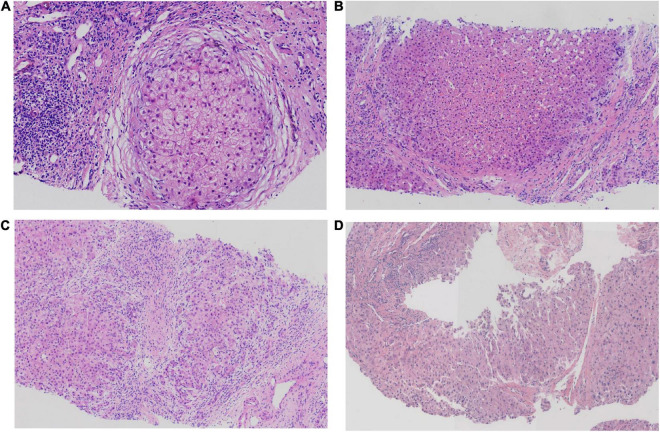
Pathologic Features of Patients with PFIC3 in liver biopsy specimens. All patients with severe liver fibrosis and even cirrhosis **(A–D)**. Panel **(A)** shows a cirrhosis nodule and an air halo in the plasma of the hepatocytes around the nodules in patient 1. Panel **(B)** shows an incomplete cirrhosis nodule and the expansion of the hepatic sinuses in patient 2. Panel **(C)** shows severe liver fibrosis and mild inflammatory infiltration accompanied by ductular proliferation in patient 3. Panel **(D)** shows liver cirrhosis in patient 4. Panels **(A–D)** show H&E-stained samples; magnification: 200× in **(A–C)** and 100× in **(D)**.

**FIGURE 2:**
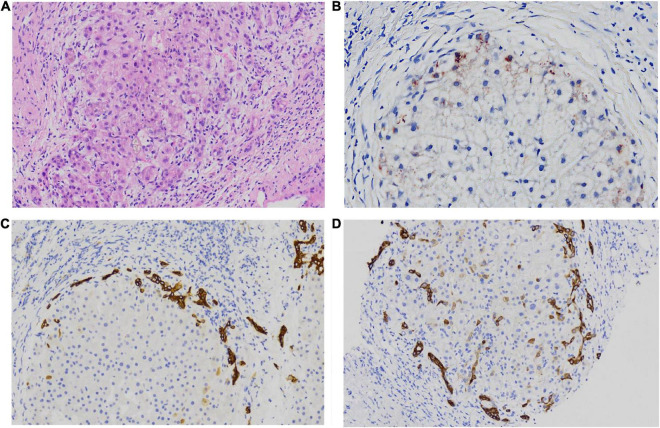
some special pathologic features of patients with PFIC3 in liver biopsy specimens. Panel **(A)** shows the presence of bile plugs in some ductules and lobules with hepatocyte cholestasis; Panel **(B)** shows orange granule deposits of copper in periportal hepatocytes (Rhodamine staining). Panel **(C)** shows immunohistochemical staining for cytokeratin 7 (ck7), which confirms the presence of interlobular duct and ductular reaction. Panel **(D)** shows the heterotopic expression of ck7 on periportal hepatocytes, indicating chronic cholestasis. Panels **(A,B)** show H&E-stained samples; Panels **(C,D)** show immunohistochemically stained samples; magnification: 200× in **(A–D)**.

**FIGURE 3:**
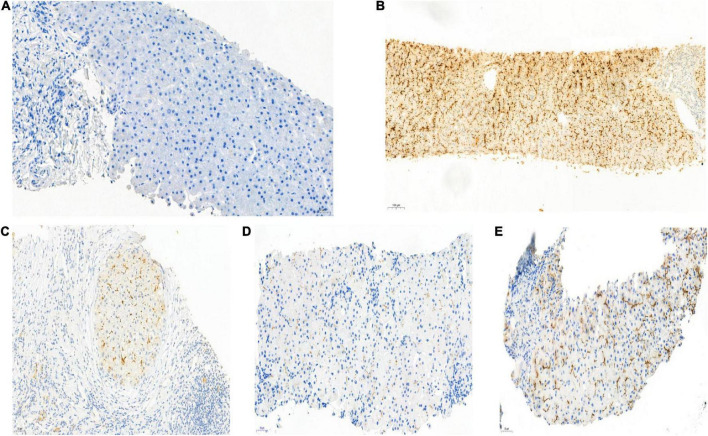
Biopsy specimens of liver samples expressing Multidrug-Resistant Protein 3. **(A)** Normal MDR3 expression is visible in liver biopsy samples from a healthy individual. **(B)** Liver biopsy specimens from Case 2: loss of MDR3 expression. **(C–E)** Liver biopsy specimens from Case 1, 3, and 4: The MDR3 staining was significantly reduced. Images showing immunohistochemical staining at a magnification of 200×.

Hepatopathological analyses revealed that all patients had extensive portal fibrosis and biliary cirrhosis (Figure 1) classified by Ishak stages 3–4 and Knodell stage 3 (17), and a missing bile duct or biliary epithelium injury was not noted. Histopathological approaches revealed an air halo in the plasma of the hepatocytes around the nodules in patient 1 (Figure 1A), an incomplete liver cirrhosis nodule and expansion of the hepatic sinuses in patient 2 (Figure 1B), inflammatory infiltration accompanied by ductular proliferation in patient 3 (Figure 1C), and liver fibrosis in patient 4 (Figure 1D). PFIC3 patients showed some pathologic features of cholestatic cirrhosis (Figure 2). Bile plugs were observed in some ductules and lobules with hepatocyte cholestasis (Figure 2A). Rhodamine staining was positive for copper particles which appeared deposited in hepatocyte plasma (Figure 2B). CK7 immunostaining within the portal tract revealed extensive proliferation of ducts (Figure 2C) and CK7 expression in some intermediate hepatocytes (Figure 2D). Bile duct reaction was found in 3/4 patients, cholestasis in all patients, portal area inflammation in 3/4 patients, and cirrhosis in all patients. Decreased expression of MDR3 was found in 3/4 patients (Table 2). In this study, hepatology analysis revealed two different patterns: one involving portal fibrosis and ductular proliferation and the other involving extensive fibrosis and biliary cirrhosis. Four of the liver specimens were examined immunohistochemically. One had absent expression of MDR3; the other three had decreased expression of MDR3 (Figure 3).

**TABLE 2:** Summary of histologic characteristics.

Histologic findings	Progressive familial intrahepatic cholestasis type 3 (PFIC3)			
	
	Case 1	Case 2	Case 3	Case 4
**Portal area**				
Lack of bile duct	No	No	No	No
Bile duct reaction	Not obvious	Obvious	Obvious	Obvious
Within the capillary bile	Obvious	Mild	Obvious	Obvious
Bile salt deposition	Obvious	Mild	Mild	Obvious
Portal area inflammation	No	Obvious	Obvious	Obvious
Lobular inflammation interface	Mild	Mild	Mild	Mild
Interfacial inflammation	Mild	Mild	Mild	Mild
Cirrhosis	Obvious	Obvious	Obvious	Obvious
MDR3 expression	Absent	Decreased	Decreased	Decreased

### Genetic features

The four patients were identified as having two types of pathogenic variants of *ABCB4*: splice site (patient 1) and missense mutations (patients 2, 3, and 4) (Table 3). *ABCB4*_ex13 (c.1560 + 2T > A) was a hybrid splicing mutation, whereas *ABCB4*_ex17 c.2177C > T (p. Pro726Leu), *ABCB4*_ex15 c.1756G > T (p. Val586Leu), and *ABCB4*_ex15 c.247IT > C (p. Val824Ala) were hybrid missense mutations.

**TABLE 3:** Patients with pathogenic *ABCB4* variants.

Patient ID	Location	Variant	Protein variant	Variant type	Zygosity	ClinVar accession
1	chr7:870 69513	c.1560 + 2T > A	*	Splicing	Hybrid mutation	Not available
2	chr7:870 53256	c.2177 C > T	p. Pro726leu	Missense	Hybrid mutation	VCV000194709.4
3	chr7:870 53256	c.2177 C > T	p. Pro726Leu	Missense	Hybrid mutation	VCV000194709.4
4	chr7:870 60857	c.1756G > T	p. Val586Leu	Missense	Hybrid mutation	Not available
5	chr7:870 47860	c.2471 T > C	p. Val824Ala	Missense	Hybrid mutation	Not available

One novel variant was identified (c.1560 + 2T > A) in patient 1; this was a hybrid splicing mutation that occurred at the classical shear site. This variation is currently not mentioned in the ClinVar database, and mutations at this site have not been reported in East Asian populations. A comprehensive judgment for suspected pathogenic mutation was made using SIFT and PolyPhen-2.

Notably, a hybrid missense mutation, c.2177C > T (p. Pro726Leu), was found in patients 2 and 3; this is a suspected pathogenic mutation according to the ClinVar database. Mutations at this site have a frequency of 0 in healthy East Asian populations. SIFT and PolyPhen-2 predicted that the function of the resultant truncated protein would be harmful.

Furthermore, c.1756G > T (p. Val586Leu) and c.2471 T > C (p. Val824Ala) were found in patients 2 and 3, respectively. These are hybrid missense mutations that have not yet been mentioned in the ClinVar database. These mutations are not known to frequently occur in East Asian populations. The function of the resultant truncated protein was predicted to be harmful by SIFT and suspected to be harmful by PolyPhen-2.

**FIGURE 4:**
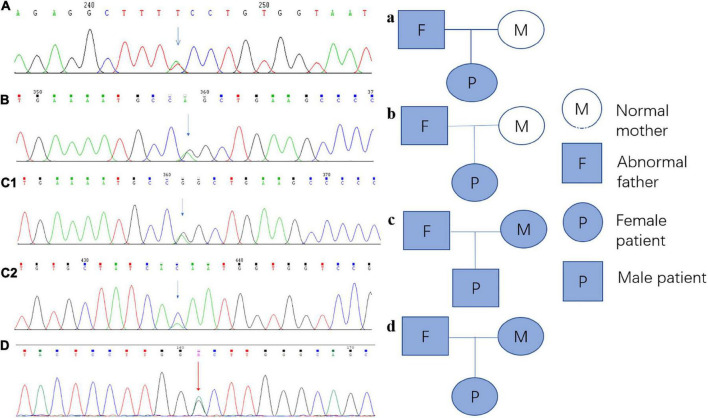
Sanger sequencing verification diagram and pedigree of the *ABCB4* variations in each patient and their parents. **(A)** Sanger sequencing verification of *ABCB4*_ex13 c.1560 + 2T > A in patient 1; a: the pedigree of patient 1 and her parents, and this variation originated from her father. **(B)** Sanger sequencing verification of *ABCB4*_ex17 c.2177C > T (p. Pro726Leu) in patient 2; b: the pedigree of patient 2 and her parents, and this variation originated from her father. **(C1)** Sanger sequencing verification of *ABCB4*_ex17 c.2177C > T (p. Pro726Leu) in patient 3, c1: the pedigree of patient 3 and his parents, and this variation originated from his mother. **(C2)** Sanger sequencing verification of *ABCB4*_ex15 c.1756G > T (p. Val586Leu) in patient 3, c2: the pedigree of patient 3 and his parents, and this variation originated from his father. **(D)** Sanger sequencing verification of *ABCB4*_ex17 c.2171T > C (p. Vol824Ala) in patient 4; d: the pedigree of patient 4, and this variation originated from her father.

Every variation in *ABCB4* genes in our patients was verified by Sanger sequencing, and a validation diagram was prepared (Figure 4). Furthermore, we traced the source of patients’ variations. Interestingly, all female patients inherited the genetic variants from their fathers; conversely, patient 3 (the only male patient) inherited one variation of the *ABCB4* gene from each parent. This also indicates that the variation in the *ABCB4* gene is dominantly inherited.

## Discussion

Clinical, pathologic, and genetic aspects of PFIC3 were studied in four Chinese patients. All patients had severe portal fibrosis, even biliary cirrhosis. All patients had similar clinical courses, with mild yellow dye in the skin and sclera and yellow urine, culminating into severe cholestasis. The *ABCB4* genetic test was conducted for all patients.

The biochemical indices of liver functions indicated that serum alanine aminotransferase (ALT), GGT, and TBA levels were continuously high at more than three times the normal values. The changes in serum GGT levels were consistent with those of ALT, TBil, and TBA levels. In the presence of high serum TBA concentration, primary bile acid synthesis disorders can be excluded. In patients with PFIC3, serum GGT activity is high, whereas in patients with PFIC1 and PFIC2, serum GGT activity is normal. A distinguishing feature of patients with PFIC3 over patients with PFIC1/PFIC2 is that the former patients are more likely to present with cholestatic jaundice later in life rather than early in infancy or childhood (18). In patients with elevated GGT levels who were suspected as having cholestasis without an obvious cause, PFIC3 should be suspected after excluding other possible causes of cholestasis (19).

Four patients underwent liver biopsies because of their prolonged jaundice, severe portal fibrosis, and abnormal laboratory values, such as increased serum DBil, GGT, and TBA; the findings and values convincingly suggested biliary cholestasis. All patients had extensive fibrosis, ductular proliferation, and biliary cirrhosis. Holostasis (bile plugs) was found in all patients. The plasma of the hepatocytes around the nodules appearing as an air halo, copper particle deposition, and positive CK7 expression of intermediate liver cells are morphological characteristics indicative of biliary cirrhosis, and all patients had these findings. In previous research, these findings have been reported to be consistent with histological changes in the liver of adult patients with PFIC3 (20). Furthermore, liver biopsies revealed ductular proliferation with mild inflammation, which is indicative of early stages of fibrosis in the portal tract. In other studies, PFIC3 has been described as having two histological patterns: the first is slight portal fibrosis and ductular proliferation and the second is extensive fibrosis and biliary cirrhosis that may present at as early as 5 years of age (21). This suggests that patients with PFIC3 can develop extensive fibrosis or bile siltation cirrhosis during adolescence or later, and the diagnosis of PFIC3 needs to be distinguished from that of other diseases, such as Wilson’s disease and primary biliary cirrhosis, on the basis of its morphological characteristics. Therefore, immunohistochemical staining was also applied in this study to assist the diagnosis.

Immunohistochemical analyses discovered absence or decreased expression of MDR3 in all patients. Because alterations in MDR3 expression are infrequent in other types of cholestatic disease, absence of or a decrease in MDR3 expression could serve as a diagnostic indicator of PFIC3. PC, a phospholipid, is transported by MDR3 through the canalicular membrane of the liver (22). Hepatocytes use MDR3 to transfer phospholipid molecules from their inner canalicular membrane to their outer canalicular membrane *via* the flippase activity. Notably, PC protects hepatocyte membranes from bile salts’ detergent activity (23–25). The activity of MDR3 is essential for bile salts to attach to PC in the canaliculus, as demonstrated in *in vitro* studies (26). Inflammation and tissue death result from the detergent action of free bile salts that can solubilize the apical membrane and hepatobiliary epithelium, leading to biliary canaliculi injury, cirrhosis, and ultimately liver failure (27). Moreover, bile with low phospholipid levels can cause cholesterol to crystallize, thus resulting in bile duct obstruction. Compared to weak or normal staining of MDR3 in patients with a reciprocal missense mutation of *ABCB4*, Jacquemin et al. observed completely negative staining of MDR3 in patients harboring a missense mutation of *ABCB4*, which results in a truncated MDR3; this is consistent with the findings of the present study. These results suggest that a diagnosis of PFIC3 requires a combination of histopathologic examination and immunohistochemical assessment of MDR3 expression. Despite this, normal immunostaining of MDR3 does not exclude genetic defects because mutations may lead to loss of function while maintaining normal synthesis. Therefore, genetic testing of *ABCB4* is essential (28).

In patients with PFIC3, genetic mutations associated with *ABCB4* occur homozygously, heterozygously, or compound heterozygously. The biallelic mutation of the *ABCB4* gene, which encodes the phospholipid flippase MDR3 on chromosome 7q21.1, contributes to the development of PFIC3 (29–31).

More than 40 different *ABCB4* gene mutations have been reported, including missense mutations, senseless mutations, deletion mutations, and insertion of small fragments of bases (32). Different types of pathogenic mutations in *ABCB4* (≥ 70% missense) lead to distinct clinical outcomes and are associated with different cumulative risks (33). Delaunay et al. devised a classification system for the various forms of these mutations. Class I includes mutations that result in defective synthesis, class II covers variations that prevent protein maturation, class III includes mutations that result in mature but defective proteins, class IV includes unstable variations, and class V includes mutations associated with unknown pathogenicity. These classifications are useful in determining potential therapies for patients depending on their genotype (14).

According to the human gene mutation database,^1^ *ABCB4* has been associated with more than 202 biliary diseases, 50 of which are related to PFIC3 (34). Besides truncated and missense mutations, nonsense mutations, deletions, and frameshift mutations occur as well. *ABCB4* gene mutation can lead to defective MDR3 expression, bile phospholipid deficiency, and persistent inflammation and fibrosis in the liver and bile tube cells caused by continuous exposure to bile salt toxicity, thus resulting in bile siltation. A nonsense mutation or deletion often manifests with severe clinical symptoms due to the severity of the mutation.

In this study, four pathogenic variants of the *ABCB4* gene (c.1560 + 2 T > A, c.2177 C > T, c.1756 G > T, and c.2471 T > C) were identified in the patients. The human gene mutation database listed the c.2177 C > T (p. Pro726Leu) hybrid missense mutation in ex17 of *ABCB4* as one that has been detected in patients with PFIC3; this mutation was found in two of our patients.^2^ Adolescent and adult patients with PFIC3 reportedly have the c.2177 C > T mutation (35–37). Patients with PFIC can also develop cholelithiasis and intrahepatic cholestasis because of mutations causing cholelithiasis and pregnancy-induced liver disease. Functional experiments indicate that the expression of mutant *ABCB4* in the bile duct has little or no effect on MDR3 protein expression, but completely impaired secretion of phosphatidyl choline results in loss of MDR3 protein expression. Mutations in the *ABCB4* gene are indicated by the absence of or a significant reduction in the expression of the MDR3 protein in liver tissues.

In addition, we identified a novel mutant of *ABCB4* in a hybrid splicing environment, namely *ABCB4*_ex13 (c.1560 + 2T > A). In accordance with the Exome Aggregation Consortium, Cambridge, Massachusetts,^3^ c. 1560 + 2T > A is a hybrid splicing mutation, which occurs at the classical shear site. This mutation may change the cutting pattern of the RNA precursor and produce abnormal splicing variants later in transcription, which subsequently affects the expression or function of the protein. The frequency of a mutation in this site in healthy East Asian population remains unknown. There is no record for this variant in ClinVar as well. It was predicted to be a pathogenic variation based on comprehensive judgment. Although the mutation was predicted to be or suspected as being harmful according to the predictive software, knowledge of the patients’ familial history and a larger sample size are needed to further investigate the correlation between the genotype and clinical phenotype.

Notably, the effect that the hybrid missense mutations c.1756 G > T and c.2471 T > C will have according to the American College of Medical Genetics and Genomics standard remains unknown. PFIC3 is caused by the autosomal recessive inheritance of the pathogenic mutation of the *ABCB4* gene (see Appendix OMIM). However, the two mutations were detected in the exon region of a single allele, which makes the patient only a carrier of the mutation. PFIC3 in this patient may be associated with this mutation; however, we did not exclude the possibility that PFIC3 in this patient could also have been caused by pathogenic mutations in non-exon regions or by other types of mutations.

A liver biopsy revealed no evidence of canalicular MDR3 expression in patient 1; this patient had a homozygous missense mutation that caused MDR3 to be defectively trafficked to the apical membrane. Regarding the other patients who exhibited canalicular expression of MDR3, albeit at a reduced level, they carried mutations that did not completely alter where the protein localized in the membrane. Treatment with ursodeoxycholic acid is normally effective for patients with reduced MDR3 expression.

Our study has some limitations, including its retrospective design, the small number of patients, and the inclusion of only East Asian patients. There is a need for larger prospective studies in Asia and globally. Patients should be recruited from different age groups, with a particular focus on the neonatal population. Furthermore, we acknowledge that family lineage testing of patients should have been conducted; during follow-up visits, we tried our best to persuade the parents to take the *ABCB4* gene test to effectively verify the pathogenicity of genetic mutations, particularly that of newly discovered mutations; however, any further information in this regard depends on the willingness of the patient’s parents to be tested. Molecular methods should be improved to elucidate the role of and mechanisms behind the new genetic mutations reported in our study.

A diagnosis of PFIC3 should be considered in patients with long-term liver dysfunction and elevated phospholipid levels presenting with severe fibrosis or bile cirrhosis. We believe that for an accurate diagnosis of PFIC3 and exclusion of other causes of cholestasis, immunohistochemistry staining of liver specimens for MDR3 and molecular genetic analysis of *ABCB4* are essential.

## Data availability statement

The data presented in this study are deposited in the NCBI BioProject repository, accession number: PRJNA876380.

## Ethics statement

The studies involving human participants were reviewed and approved by Ethical Committee of The Affiliated Hospital of Hangzhou Normal University. Written informed consent for participation was not required for this study in accordance with the national legislation and the institutional requirements.

## Author contributions

JC wrote the manuscript. LG acquired clinical information of the patient. XM analyzed the genetic results of the patients. XW and JZ performed the pathological sections and histochemical staining. WY designed and revised the manuscript. All authors read and approved the final manuscript.

## Footnotes

1
http://www.hgmd.org/

2
https://www.ncbi.nlm.nih.gov/clinvar/variation/194709

3 http://exac.broadinstitute.org, accessed September 2017.

## References

[1] Morotti RA Suchy FJ Magid MS. Progressive familial intrahepatic cholestasis (PFIC) type 1, 2, and 3: a review of the liver pathology findings. *Semin Liver Dis.* (2011) 31:3–10. 10.1055/s-0031-1272831 21344347

[2] Vitale G Gitto S Vukotic R Raimondi F Andreone P. Familial intrahepatic cholestasis: new and wide perspectives. *Dig Liver Dis.* (2019) 51:922–33. 10.1016/j.dld.2019.04.013 31105019

[3] Srivastava A. Progressive familial intrahepatic cholestasis. *J Clin Exp Hepatol.* (2014) 4:25–36. 10.1016/j.jceh.2013.10.005 25755532 PMC4017198

[4] Lang T Haberl M Jung D Drescher A Schlagenhaufer R Keil A Genetic variability, haplotype structures, and ethnic diversity of hepatic transporters MDR3 (ABCB4) and bile salt export pump (ABCB11). *Drug Metab Dispos.* (2006) 34:1582–99. 10.1124/dmd.105.008854 16763017

[5] Davit-Spraul A Fabre M Branchereau S Baussan C Gonzales E Stieger B ATP8B1 and ABCB11 analysis in 62 children with normal gamma-glutamyl transferase progressive familial intrahepatic cholestasis (PFIC): phenotypic differences between PFIC1 and PFIC2 and natural history. *Hepatology.* (2010) 51:1645–55. 10.1002/hep.23539 20232290

[6] Mullenbach R Lammert F. An update on genetic analysis of cholestatic liver diseases: digging deeper. *Dig Dis.* (2011) 29:72–7. 10.1159/000324137 21691109

[7] Sharma A Poddar U Agnihotry S Phadke SR Yachha SK Aggarwal R. Spectrum of genomic variations in Indian patients with progressive familial intrahepatic cholestasis. *BMC Gastroenterol.* (2018) 18:107. 10.1186/s12876-018-0835-6 29973134 PMC6032793

[8] Amirneni S Haep N Gad MA Soto-Gutierrez A Squires JE Florentino RM. Molecular overview of progressive familial intrahepatic cholestasis. *World J Gastroenterol.* (2020) 26:7470–84. 10.3748/wjg.v26.i47.7470 33384548 PMC7754551

[9] Ganne-Carrié N Baussan C Grando V Gaudelus J Cresteil D Jacquemin E. Progressive familial intrahepatic cholestasis type 3 revealed by oral contraceptive pills. *J Hepatol.* (2003) 38:693–4. 10.1016/s0168-8278(03)00049-7 12713886

[10] Degiorgio D Crosignani A Colombo C Bordo D Zuin M Vassallo E ABCB4 mutations in adult patients with cholestatic liver disease: impact and phenotypic expression. *J Gastroenterol.* (2016) 51:271–80. 10.1007/s00535-015-1110-z 26324191

[11] Reichert MC Lammert F. ABCB4 gene aberrations in human liver disease: an evolving spectrum. *Semin Liver Dis.* (2018) 38:299–307. 10.1055/s-0038-1667299 30357767

[12] Wei G Cao J Huang P An P Badlani D Vaid KA Synthetic human ABCB4 mRNA therapy rescues severe liver disease phenotype in a BALB/c.Abcb4(-/-) mouse model of PFIC3. *J Hepatol.* (2021) 74:1416–28. 10.1016/j.jhep.2020.12.010 33340584 PMC8188846

[13] Zhang W Lin R Lu Z Sheng H Xu Y Li X Phenotypic and molecular characteristics of children with progressive familial intrahepatic cholestasis in South China. *Pediatr Gastroenterol Hepatol Nutr.* (2020) 23:558–66. 10.5223/pghn.2020.23.6.558 33215027 PMC7667226

[14] Delaunay JL Durand-Schneider AM Dossier C Falguières T Gautherot J Davit-Spraul A A functional classification of ABCB4 variations causing progressive familial intrahepatic cholestasis type 3. *Hepatology.* (2016) 63:1620–31. 10.1002/hep.28300 26474921

[15] European Association for the Study of the Liver. EASL Clinical Practice Guidelines: management of cholestatic liver diseases. *J Hepatol.* (2009) 51:237–67. 10.1016/j.jhep.2009.04.009 19501929

[16] Richards S Aziz N Bale S Bick D Das S Gastier-Foster J Standards and guidelines for the interpretation of sequence variants: a joint consensus recommendation of the American College of Medical Genetics and Genomics and the Association for Molecular Pathology. *Genet Med.* (2015) 17:405–24. 10.1038/gim.2015.30 25741868 PMC4544753

[17] Goodman ZD. Grading and staging systems for inflammation and fibrosis in chronic liver diseases. *J Hepatol.* (2007) 47:598–607. 10.1016/j.jhep.2007.07.006 17692984

[18] Davit-Spraul A Gonzales E Baussan C Jacquemin E. The spectrum of liver diseases related to ABCB4 gene mutations: pathophysiology and clinical aspects. *Semin Liver Dis.* (2010) 30:134–46. 10.1055/s-0030-1253223 20422496

[19] Poupon R Rosmorduc O Boelle PY Chretien Y Corpechot C Chazouilleres O Genotype-phenotype relationships in the low-phospholipid-associated cholelithiasis syndrome: a study of 156 consecutive patients. *Hepatology.* (2013) 58:1105–10. 10.1002/hep.26424 23533021

[20] Elderman JH Ter Borg PC Dees J Dees A. Pregnancy and ABCB4 gene mutation: risk of recurrent cholelithiasis. *BMJ Case Rep.* (2015) 2015:bcr2014206919. 10.1136/bcr-2014-206919 25612754 PMC4307091

[21] Olsen JA Alam A Kowal J Stieger B Locher KP. Structure of the human lipid exporter ABCB4 in a lipid environment. *Nat Struct Mol Biol.* (2020) 27:62–70. 10.1038/s41594-019-0354-3 31873305

[22] Reichert MC Hall RA Krawczyk M Lammert F. Genetic determinants of cholangiopathies: Molecular and systems genetics. *Biochim Biophys Acta Mol Basis Dis.* (2018) 1864:1484–90. 10.1016/j.bbadis.2017.07.029 28757171

[23] Dixon PH Williamson C. The pathophysiology of intrahepatic cholestasis of pregnancy. *Clin Res Hepatol Gastroenterol.* (2016) 40:141–53. 10.1016/j.clinre.2015.12.008 26823041

[24] Khabou B Durand-Schneider AM Delaunay JL Ait-Slimane T Barbu V Fakhfakh F Comparison of in silico prediction and experimental assessment of ABCB4 variants identified in patients with biliary diseases. *Int J Biochem Cell Biol.* (2017) 89:101–9. 10.1016/j.biocel.2017.05.028 28587926

[25] Gunaydin M Bozkurter Cil AT. Progressive familial intrahepatic cholestasis: diagnosis, management, and treatment. *Hepat Med.* (2018) 10:95–104. 10.2147/HMER.S137209 30237746 PMC6136920

[26] Prescher M Smits SHJ Schmitt L. Stimulation of ABCB4/MDR3 ATPase activity requires an intact phosphatidylcholine lipid. *J Lipid Res.* (2020) 61:1605–16. 10.1194/jlr.RA120000889 32917728 PMC7707170

[27] Jacquemin E De Vree JM Cresteil D Sokal EM Sturm E Dumont M The wide spectrum of multidrug resistance 3 deficiency: from neonatal cholestasis to cirrhosis of adulthood. *Gastroenterology.* (2001) 120:1448–58. 10.1053/gast.2001.23984 11313315

[28] Keitel V Burdelski M Warskulat U Kuhlkamp T Keppler D Haussinger D Expression and localization of hepatobiliary transport proteins in progressive familial intrahepatic cholestasis. *Hepatology.* (2005) 41:1160–72. 10.1002/hep.20682 15841457

[29] Groen A Romero MR Kunne C Hoosdally SJ Dixon PH Wooding C Complementary functions of the flippase ATP8B1 and the floppase ABCB4 in maintaining canalicular membrane integrity. *Gastroenterology.* (2011) 141:e1921–4. 10.1053/j.gastro.2011.07.042 21820390

[30] Guyot C Stieger B. Interaction of bile salts with rat canalicular membrane vesicles: evidence for bile salt resistant microdomains. *J Hepatol.* (2011) 55:1368–76. 10.1016/j.jhep.2011.04.014 21703191

[31] Park HJ Kim TH Kim SW Noh SH Cho KJ Choi C Functional characterization of ABCB4 mutations found in progressive familial intrahepatic cholestasis type 3. *Sci Rep.* (2016) 6:26872. 10.1038/srep26872 27256251 PMC4891722

[32] Hochrath K Ehnert S Ackert-Bicknell CL Lau Y Schmid A Krawczyk M Modeling hepatic osteodystrophy in Abcb4 deficient mice. *Bone.* (2013) 55:501–11. 10.1016/j.bone.2013.03.012 23545228 PMC4075965

[33] Saleem K Cui Q Zaib T Zhu S Qin Q Wang Y Evaluation of a novel missense mutation in ABCB4 gene causing progressive familial intrahepatic cholestasis type 3. *Dis Markers.* (2020) 2020:6292818. 10.1155/2020/6292818 32626542 PMC7315263

[34] Gautherot J Delautier D Maubert MA Ait-Slimane T Bolbach G Delaunay JL Phosphorylation of ABCB4 impacts its function: insights from disease-causing mutations. *Hepatology.* (2014) 60:610–21. 10.1002/hep.27170 24723470

[35] Lincke CR Smit JJ van der Velde-Koerts T Borst P. Structure of the human MDR3 gene and physical mapping of the human MDR locus. *J Biol Chem.* (1991) 266:5303–10. 10.1016/s0021-9258(19)67788-4 2002063

[36] Degiorgio D Colombo C Seia M Porcaro L Costantino L Zazzeron L Molecular characterization and structural implications of 25 new ABCB4 mutations in progressive familial intrahepatic cholestasis type 3 (PFIC3). *Eur J Hum Genet.* (2007) 15:1230–8. 10.1038/sj.ejhg.5201908 17726488

[37] Stenson PD Mort M Ball EV Shaw K Phillips A Cooper DN. The Human Gene Mutation Database: building a comprehensive mutation repository for clinical and molecular genetics, diagnostic testing and personalized genomic medicine. *Hum Genet.* (2014) 133:1–9. 10.1007/s00439-013-1358-4 24077912 PMC3898141

